# Effects Associated with Nanostructure Fabrication Using In Situ Liquid Cell TEM Technology

**DOI:** 10.1007/s40820-015-0054-4

**Published:** 2015-07-28

**Authors:** Xin Chen, Lihui Zhou, Ping Wang, Hongliang Cao, Xiaoli Miao, Feifei Wei, Xia Chen

**Affiliations:** 1 Shanghai Key Laboratory of Advanced Polymeric Materials, and Key Laboratory for Ultrafine Materials of Ministry of Education, School of Materials Science and Engineering, East China University of Science and Technology, 130 Meilong Road, Shanghai, 200237 People’s Republic of China; 2 East China University of Science and Technology, 130 Meilong Road, Shanghai, 200237 People’s Republic of China; 3 State Key Laboratory of Bioreactor Engineering, Biomedical Nanotechnology Center, East China University of Science and Technology, Shanghai, 200237 People’s Republic of China; 4 State Key Laboratory of Functional Materials for Informatic, Shanghai Institute of Microsystem and Information Technology, Chinese Academy of Sciences, 865 Changning Road, Shanghai, 200050 People’s Republic of China

**Keywords:** Electron-beam-induced deposition, In situ TEM, Nanostrucutre, Semiconductor, Nanolithography

## Abstract

We studied silicon, carbon, and SiC_*x*_ nanostructures fabricated using liquid-phase electron-beam-induced deposition technology in transmission electron microscopy systems. Nanodots obtained from fixed electron beam irradiation followed a universal size versus beam dose trend, with precursor concentrations from pure SiCl_4_ to 0 % SiCl_4_ in CH_2_Cl_2_, and electron beam intensity ranges of two orders of magnitude, showing good controllability of the deposition. Secondary electrons contributed to the determination of the lateral sizes of the nanostructures, while the primary beam appeared to have an effect in reducing the vertical growth rate. These results can be used to generate donut-shaped nanostructures. Using a scanning electron beam, line structures with both branched and unbranched morphologies were also obtained. The liquid-phase electron-beam-induced deposition technology is shown to be an effective tool for advanced nanostructured material generation.

## Introduction

Focused beams of electrons and ions are valuable tools for making micro–nanostructures, which have great potential in such future applications as integrated circuits [1]. Among the various focused beam methods, electron-beam-induced deposition (EBID) is a powerful technique that has attracted widespread interest in recent decades for nanofabrication applications [2–7]. In the last decade, benefitting from the development of thin film microfabrication technology, liquid cell electron microscopy has drawn much international attention [8–12]; and currently, liquid-phase precursor materials can be used instead of traditional gas-phase precursors for EBID [13–15].

Currently, both scanning electron microscope (SEM) and transmission electron microscope (TEM) have been used in liquid-phase electron-beam-induced deposition (LP-EBID) research. Among these two technologies, the TEM approach offers higher imaging resolution and thus provides a better tool for in situ study of the material growth behavior during EBID. Using this technique, researchers have deposited many different nanomaterials, such as silver nanoaggregates, Pt, Pd, and PbS nanomaterials [16–21]. In addition to the relatively irregular-shaped nanomaterials, Grogan et al. have demonstrated direct writing of nanoscale Au letters using LP-EBID [22], and we have demonstrated controlled deposition of Si nanodots, SiC_*x*_ nanodots, and SiC_*x*_ nanolines using this technology [15, 23].

Although progress has been made, LP-EBID is still a very young technology compared with the traditional EBID, and there is still a need to find methods to controllably develop relatively complicated nanostructures with the LP-EBID method.

In this paper, based on the SiC_*x*_ material system, we address some effects associated with nanomaterial formation using LP-EBID, which will be helpful for controllably forming complicated nanostructures with this technology.

## Experimental

A homemade in situ liquid TEM cell was used for the experiment. Liquid precursors were enclosed between two Si_3_N_4_ window grids in the in situ cell. The details of the in situ cell structure have been previously reported [15, 23]. Metallic thin film spacers of ~100 nm were formed on one of the grids to limit the minimum space between the substrates; however, the typical separation between the Si_3_N_4_ windows was generally larger and also varied from place to place because of the Si_3_N_4_ window deformation resulting from the clamping pressure. The EB was focused on the Si_3_N_4_ windows to induce the breaking up of the precursor molecules and the deposition of the nanomaterials. Because of the bowing up deformation, the separation of the two Si_3_N_4_ windows can be of the order of 10 µm [15]; thus, the focus conditions for the two windows are usually different, and the focused beam exposure/nanostructure development observation was usually performed on the top substrate only.

Si_3_N_4_ window grids with window thicknesses of 50 or 200 nm were purchased from Ted Pella, Inc. (Redding, CA, USA). SiCl_4_ in CH_2_Cl_2_ solutions of different concentrations (1 M, 4 M, and pure SiCl_4_) was prepared by mixing 1 M SiCl_4_ in CH_2_Cl_2_ solution (0.95–1.10 M, Alfa Aesar, Ward Hill, MA, USA) and a pure SiCl_4_ (99.998 % purity, Alfa Aesar, Ward Hill, MA, USA). CH_2_Cl_2_ (99.5 %) from Sinopharm Chemical Reagent Co. Ltd. of Shanghai (Shanghai, People’s Republic of China) was also used for comparison (denoted as 0 M SiCl_4_ concentration). An argon-filled Mbraun Labstar (1950/780) dry glovebox workstation (M. Braun Incorporated, Stratham, NH, USA) was used for the precursor preparation and loading procedure.

A JEOL 2100 Cryo TEM, a JEOL JEM 2100 TEM, and a JEOL 2010 LaB6 TEM (JEOL Ltd., Tokyo, Japan) were used for the LP-EBID study, all operated under a 200 kV electron acceleration voltage, with focused beam sizes of approximately 30 nm and with the beam currents calibrated. After the liquid cell was dissembled and the SiC_*x*_ deposited grids were taken out, a FEI Dual Beam 235 dual-beam focused ion beam scanning electron microscope (FEI, Hillsboro, OR, USA) was used to fabricate Pt electrodes on to the SiC_*x*_ nanostructures. The topography of these nanostructures was characterized with an Asylum Research MFP-3D atomic force microscope (AFM) (Asylum Research, an Oxford Instruments Company, Santa Barbara, CA, USA).

## Results and Discussion

### NanoDots and NanoLine Structures Prepared with LP-EBID

**Fig. 1:**
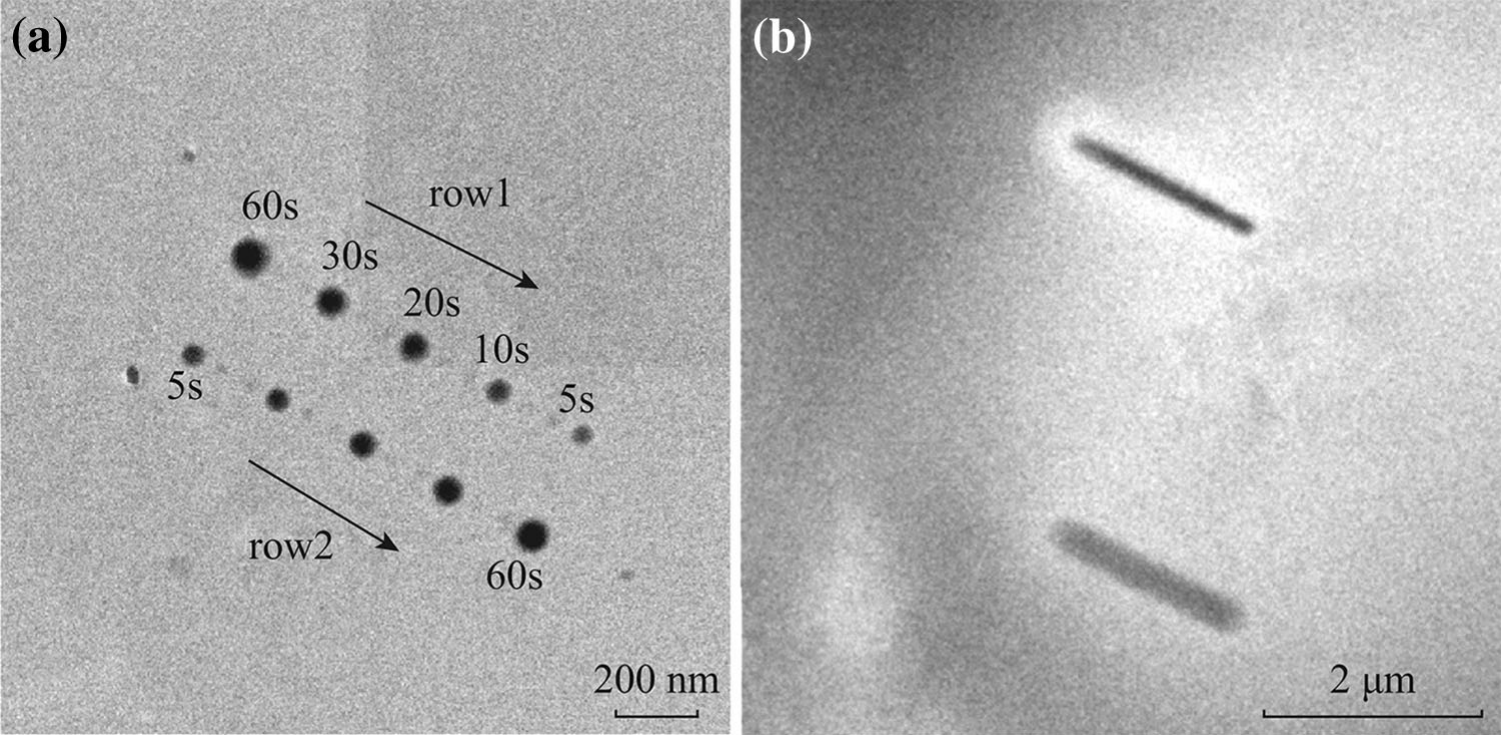
SiC_*x*_ nanodots and nanoline structures formed with LP-EBID. The precursor was 1 M SiCl_4_ solution. **a** Nanodots formed with a beam current of 0.28 nA, and **b** nanoline structures formed with scanning beams of 0.15 and 0.3 nA

First, we tested the LP-EBID method by depositing nanodots and nanoline structures [15, 23]. By focusing the electron beam on the Si_3_N_4_ window for various lengths of time, we obtained nanodots of different sizes. A Faraday cup measurement has been used for the beam current calibration [15]. As shown in Fig. 1a, using the 1 M precursor solution, we obtained SiC_*x*_ nanodots of different sizes. The focused electron beam current was 0.28 nA, and the exposure time varied from 5 to 60 s, resulting in dot sizes from ~50–60 to ~80–90 nm. Right after row 1 was deposited, row 2 was deposited subsequently in the region. Row 1 shows an array of nanodots deposited with the longer exposure time first, and row 2 was deposited with the shorter exposure time first. In addition to an increase in the lateral dot size, the dots also became darker with longer exposure time, indicating a three-dimensional (3-D) size increase. The dot sizes were relatively unaffected by the exposure sequence and showed relatively clear boundaries, indicating minimal proximity effects [24] and good size controllability. In aqueous solution systems, the beam exposure history showed strong influence on the material growth behavior, resulting in a significant reduction of nanoparticle growth in the subsequent experiments due to the depletion of precursor in the solution [20, 25]. This phenomenon is not observed in our experiment, as our precursors are the main body of the liquid in the liquid cell.

By scanning the focused beam across the SiN_*x*_ window, we also deposited various line structures. Figure 1b shows the TEM image of two line structures that have been created with a beam scanning speed of 60 nm s^−1^ and a scan time of 120 s. The wider line was deposited with a beam intensity of ~0.3 nA, which resulted in a line width of ~300 nm and length of ~2000 nm; the narrower line was deposited with a beam intensity of ~0.15 nA, which resulted in a line width of ~150 nm and length of ~1800 nm. The brightness contrast in the background suggests that there might be liquid layer thickness variation and/or bubble existence in the region.

The above focused beam position change and beam scanning have been achieved by adjusting the *x*–*y* shift knobs of the TEM instrument. For Fig. 1a, to get a good dot position separation, 3 coarse shift steps have been made for each beam position change, and the transient beam left some much smaller nanodots (~20–30 nm sized) between the labeled larger dots. For Fig. 1b, under the fine beam position adjust mode, each shift step only yielded a very tiny beam position change, which was used to mimic a continuous scanning beam.

Image resolution is one factor under consideration in liquid cell TEM research. Resolutions better than 1 nm have been obtained using our liquid cell system [26], and the observation of nanoparticles with diameters of 1.4 nm was reported in the literature with liquid thickness up to 3.3 µm [27]. The scattering of the electron beam in a thick layer of liquid can cause some deterioration in image resolution [27], depending on the studied liquid materials and instrument factors. Bubbles may form in the liquid cell and significantly reduce the local liquid layer thickness in the cell [22, 23]. The image resolution from Fig. 1a is about 10 nm, and the resolution from Fig. 1b appears a bit poorer, possibly because of the different local liquid layer thicknesses.

### NanoDot Size Relationship with Deposition Parameters

**Fig. 2:**
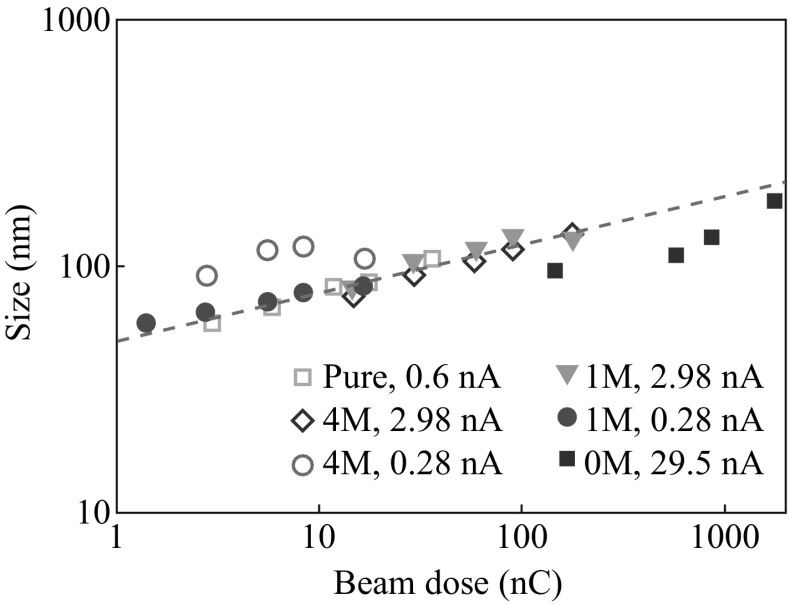
Si, SiC_*x*_, and C nanoparticle size relationship with electron beam dose, using precursors of various concentrations and various beam currents

We deposited nanodots using various precursors, with various beam intensities and deposition times. The dot size relationship with beam dose is shown in Fig. 2. As can be seen, although the precursor concentration varied from 0 M SiCl_4_ in CH_2_Cl_2_ to 100 % (pure) SiCl_4_ and the beam current changed 100-fold, there appears to be a universal trend of nanodot size growth, roughly following a single straight line in the log–log plot, indicating very good size controllability with this LP-EBID method. This linear trend indicates there is a power law growth behavior with the dot lateral size *d* proportional to *D* ^*n*^, where *D* is the beam dose. (The nanodot size dependance with time also showed the linear trend in the log–log plot, but with the higher beam current deposited dots lying toward the shorter time side, and lower current deposited dots lying toward the longer time side, without forming a single universal trend line.) The fact that the dot size was determined by the electron dose from the exposure, but relatively unaffected by the beam intensity under a certain dose condition, suggests that incident electrons were interacting with the sample independently from each other in determining the lateral size of the nanodots. Secondary electrons have been associated with the nanomaterial growth in LP-EBID [15]. The secondary electron generation rate might be expected to decrease quickly with lateral distance from the beam center, resulting in a much smaller lateral growth rate at larger *d*, and thus a small *n*-value, and dot growth might be biased toward the vertical direction. The yield of Si and C reduction from the EB irradiation may be similar during the deposition; thus, the SiCl_4_ concentration change mostly resulted in a composition shift, but did not affect the lateral size of the resulting nanodots under the same accumulated EB dose [23]. There are some relatively scattered data such as the 4 M, 0.28 nA and the 0 M, 29.5 nA, in which the data above the trend line could be due to some non-ideal beam conditions and the data below the trend line could be related to some growth rate reducing effect under the higher primary beam current, and/or there could be some minor growth rate difference between Si and C.

Note our deposited dots are localized at the beam exposed location, with well-defined shapes. This suggests that the growth was not governed by random walk diffusion [15], which tends to result in diffusion-limited aggregation of fractal dimensioned structures. On the other hand, a simplified reaction–diffusion model has been proposed, which appeared to explain our nanodot growth behavior [22, 23].

It should be noted that the linear size versus dose trend in the log–log plot may not continue to be valid for larger beam intensity ranges. For smaller beam intensities, there could be a spatial distribution of secondary electron emission that would limit further nanodot size reduction, while for stronger beam intensities, there could be such issues as the instrumental beam focusing capability and the precursor availability that will affect the size and shape of the deposited material, which need to be further examined.

### Donut-Shaped Nanostructure Development

Although the lateral size appeared to be relatively unaffected by beam intensity under a certain beam dose, in our earlier work, we reported that the volume growth rate was reduced with a higher beam current [15]. This is a result of the reduced growth rate in the thickness direction, especially in the beam center region. Secondary electrons have been suggested to be the main driver for nanodot growth, while primary electrons have been suggested to have an effect in reducing the growth rate [15].

Normally, the focused beam intensity should be the highest in the center region and fall off quickly in the radius direction; thus, the growth rate reduction effect should be the strongest in the center, but would become negligible in the outer regions. As a result, the primary beam strongly reduces the growth rate in the central region, but has almost no effect on the lateral size of the particles.

**Fig. 3:**
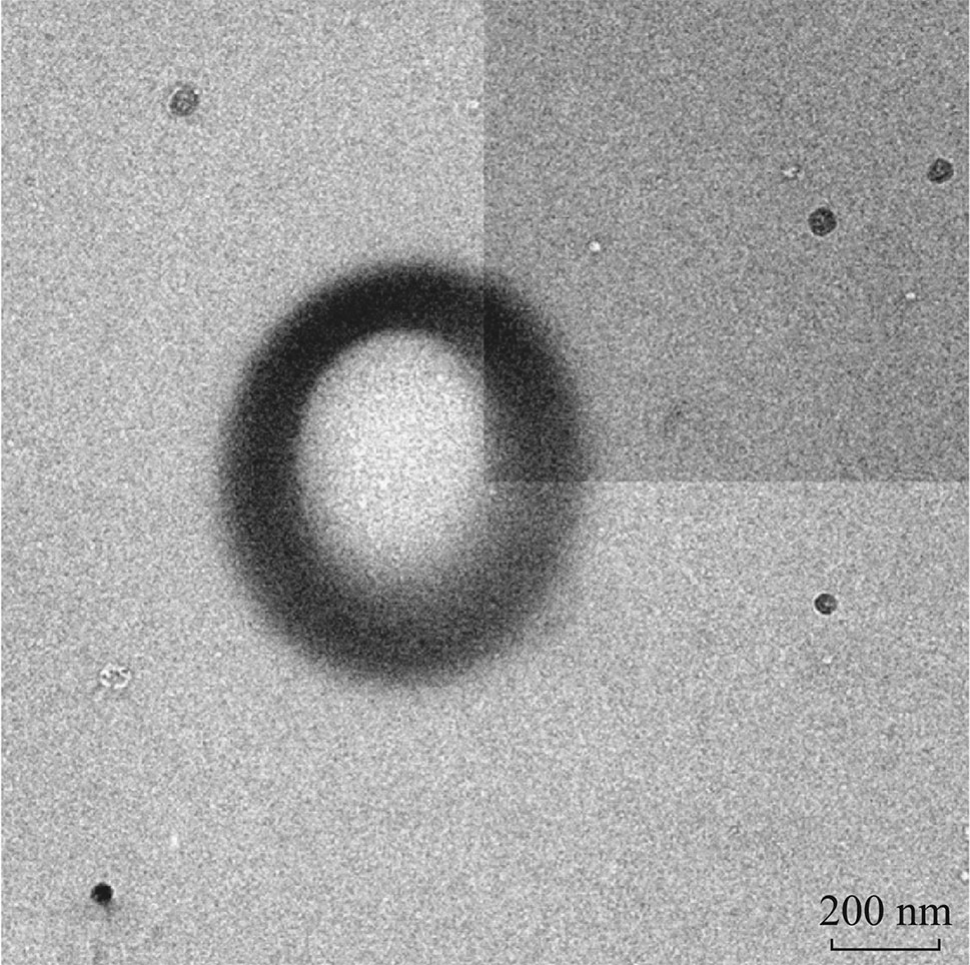
A donut-shaped SiC_*x*_ structure developed with LP-EBID, using a beam current of 36.9 nA and exposure time of 180 s

To further check the effect on growth rate of the primary beam, we used an even higher beam intensity of 36.9 nA; as can be seen in Fig. 3, the growth in the center region basically stopped, forming a donut-shaped particle. This demonstrates that a deeper understanding of the growth mechanism can help people to develop materials with more sophisticated structures. With such a high beam current, the instrumental beam focus conditions were less than ideal and the deposition was less round, which may be related to beam astigmatism. Reliably creating donut-shaped nanostructures under less extreme beam conditions remains to be studied.

It will be interesting to compare our data with the recent work of Schneider et al. [28], in which, gold nanorods in water have been observed under electron beam irradiation. In that aqueous system, the gold particle size change with time was also related with the electron beam intensity; however, in that system, the gold particles were observed to grow under stronger beam, not grow or even reduce in size under weaker beam.

In our system, the stronger beam may cause the decomposition of the precursor and the generation of chlorine gas [15], which, along with the evaporation of the precursor, will reduce the local precursor supply and growth rate. Besides, our deposition was induced with focused beam, which is expected to have much higher area specific intensity than the spread beam in the experiment of Schneider et al. Why our system showed an opposite growth trend associated with different beam intensities from the aqueous system remains to be studied.

### Branched NanoLine Structure Development

**Fig. 4:**
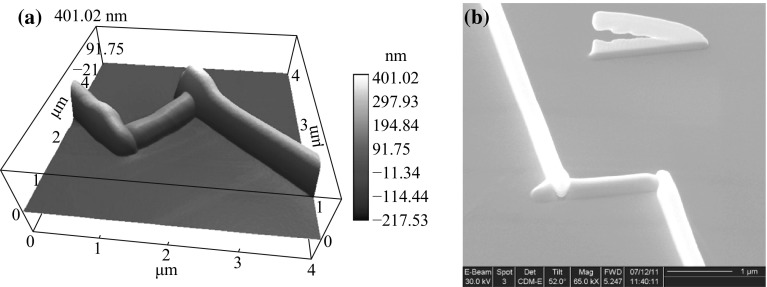
Different SiC_*x*_ 3-D nanostructures developed with LP-EBID. **a** AFM of a micro device with Pt electrodes made by a focused ion beam scanning electron microscope on both ends of a SiC_*x*_ nanoline, and **b** an SEM image of larger area taken at a 52° tilt angle showing a branched 3-D structure for the* narrower line* in Fig. 1b

In addition to nanostructures deposited under a fixed electron beam, we further studied line structures deposited using a scanning beam. We dissembled the cell and examined the nanoline structures of Fig. 1b. For the wider line, ex situ imaging showed a smooth and relatively uniform nanoline, which we were able to further process by depositing electrode wires on both ends of it to form a micro device. An AFM image of the device is shown in Fig. 4a. The narrower line appeared as a branched structure in a tilted SEM image, which is shown in the top part of Fig. 4b. Note that the relative positions of the two line structures are mirror images of Fig. 1b because the liquid cell is now opened, with the SiN_x_ window side of the chip flipped over to the top.

**Fig. 5:**
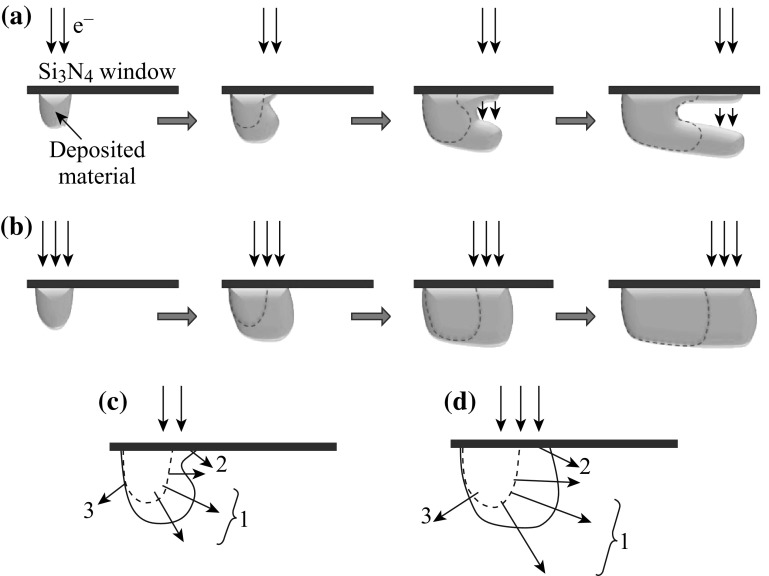
A schematic for 3-D structural development in LP-EBID. **a** Branched nanostructure development under relatively low beam intensity, **b** unbranched nanoline development under relatively high beam intensity, **c** and **d** secondary electron intensity distribution and nanostructure evolution under low and high beam intensity

A schematic for the 3-D structural development is shown in Fig. 5. Secondary electrons contribute to the reduction of the precursor and cause material growth on a surface. As shown in Fig. 5a, in the first stage, the primary electron beam (demonstrated as downward pointing arrows) caused some secondary electron emission from the substrate and some initial material growth under the Si_3_N_4_ window in the liquid chamber. Then, in the second stage, as the beam penetrates through the initially deposited material, the newly generated secondary electron emission and the growth center shifted lower than the Si_3_N_4_ window surface level; accompanied by the beam scan, the majority of the laterally grown material was located lower than the Si_3_N_4_ substrate. At the same time, there was also a small amount of secondary electron emission and material deposition on the Si_3_N_4_ window surface, thus forming a neck at the growth front. In the following stages, while the material growth on the Si_3_N_4_ window surface continued, following the scanning beam, the penetrating beam also passed through the lower side of the growth front, generating secondary electrons to continue the growth, so that the neck became deeper and deeper, finally forming two line branches. During this process, the preferred secondary emission and growth at the lower side had continuous effects, so that the separation of the lower branch from the Si_3_N_4_ window became larger and larger. There could be some proximity effects that reduce the upper branch vertical growth speed to avoid further branch generations in our experiment, which remain to be further studied.

Figure 5b shows that, under a stronger beam, although the growth center shifted downwards after the initial deposition, a larger amount of material was deposited during the same time and filled the space between the deposited material and the Si_3_N_4_ substrate, avoiding neck formation. Thus, with increasing amounts of time, the deposited line structure never actually separated from the substrate, resulting in an unbranched line compared with the case of Fig. 5a.

Figure 5c and d schematically shows the secondary electron intensity distribution and nanostructure evolution under different beam intensities. In Fig. 5c, the three arrows labeled as group 1 represent secondary electron emission from the initially grown nanostructure, with arrow lengths represent the local secondary electron intensities, with spatial distribution related to the nanostructure in the region, which is expected to be stronger toward the lower side, thus resulting a stronger local growth speed toward the lower region, away from the window. Local availability of precursor and reduced atoms may also contribute to this biased growth. Arrow 2 indicates secondary electron emission from the substrate, which causes the local material growth on the window surface and the neck formation in the growth front. The local availability of precursor and reduced atoms may have decreased the growth speed on this local window surface, and the very thin window thickness might resulted in a lower chance of electron collision and secondary electron generation here compared to the lower region correlated to arrow group 1. Arrow 3 indicates secondary electron emission away from the beam moving direction, which also causes local material growth, but the growth is not sustained as the distance with beam increases with time. Figure 5d shows, although there is still the growth bias, as the secondary electron intensities and growth speeds are higher, the possible gap between regions of 1 and 2 is filled, avoiding the neck formation.

Similar branched structures have also been reported in the literature for traditional EBID materials [3]; however, the detailed structural development behaviors are not the same. In the traditional EBID case, the material growth appears to be more concentrated at the beam location, while in our LP-EBID case, the material growth tends to spread out into larger volumes. Additionally, the traditional EBID observations have been made with materials deposited on the substrate surface facing the beam, while our branched material formation happened on the opposite side of the substrate.

## Summary and Conclusions

Silicon, carbon, and SiC_*x*_ nanostructures fabricated using LP-EBID in TEM were studied. Nanodots deposited from precursors with various SiCl_4_/CH_2_Cl_2_ concentration ratios appeared to follow a universal lateral size versus beam dose trend. In addition to secondary electrons, the primary beam intensity is further important for determining the detailed structure of the deposited material: a fixed beam with lower intensity resulted in solid centered nanodots, while deposition under a strong beam resulted in hollow-centered nanostructures; a scanning beam with lower intensity tends to generate branched line structures, while deposition under a strong scanning beam is effective in creating unbranched nanolines on the Si_3_N_4_ substrate. LP-EBID can be an effective tool in developing advanced future materials with different types of nanostructures, and deeper understanding of the primary and secondary electron effects during the growth procedure will be important for achieving this goal.
